# Modelling Blood Flow and Metabolism in the Piglet Brain During Hypoxia-Ischaemia: Simulating pH Changes

**DOI:** 10.1007/978-1-4614-7411-1_44

**Published:** 2013-03-25

**Authors:** Tharindi Hapuarachchi, Tracy Moroz, Alan Bainbridge, David Price, Ernest Cady, Esther Baer, Kevin Broad, Mojgan Ezzati, David Thomas, Xavier Golay, Nicola J. Robertson, Ilias Tachtsidis

**Affiliations:** 00441 CoMPLEX, University College London, London, UK; 00442 Medical Physics and Bioengineering, University College London, London, UK; 00443 Department of Medical Physics and Bioengineering, University College London, London, UK; 00444 Institute for Women’s Health, University College London, London, UK; 00445 Institute of Neurology, University College London, London, UK

**Keywords:** Magnetic Resonance Spectroscopy, Flavin Adenine Dinucleotide, Flavin Adenine Dinucleotide, Neonatal Brain, Carotid Artery Occlusion

## Abstract

We describe the extension of a computational model of blood flow and metabolism in the piglet brain to investigate changes in neonatal intracellular brain pH during hypoxia-ischemia (HI). The model is able to simulate near-infrared spectroscopy (NIRS) and magnetic resonance spectroscopy (MRS) measurements obtained from HI experiments conducted in piglets. We adopt a method of using ^31^P-MRS data to estimate of intracellular pH and compare measured pH and oxygenation with their modelled counterparts. We show that both NIRS and MRS measurements are predicted well in the new version of the model.

## Introduction

Experimental studies have shown a shift in brain pH following hypoxia-ischemia (HI) – the deprivation of oxygen supply [1]. HI is a major cause of perinatal brain injury [2]. Modest changes in pH can result in alterations to protein structures and therefore affect the function of membrane channels and enzymes crucial to many vital cellular functions.

Piglets are often used as models of human neonates in experimental studies involving anoxic and/or hypoxic and ischaemic insults. In order to investigate HI and to better understand the results from these experiments, we have built a computational model of blood flow and metabolism in the neonatal piglet brain (BrainPiglet) [2]. This model is an adaptation and extension of an earlier model of the adult human brain [3]. The model is used to simulate near-infrared spectroscopy (NIRS) and magnetic resonance spectroscopy (MRS) data – two non-invasive methods used to monitor brain tissue oxygenation, haemodynamics and metabolism during HI experiments. We have recently extended the model further, by simulating carotid artery occlusion [4] and intracellular brain pH (BrainPiglet v2). In this chapter, we (i) describe the main dynamics of intracellular H^+^ ions incorporated in order to model pH, (ii) explain the methods used to obtain an estimate of brain pH from ^31P-MRS^ measurements and (iii) validate the model by comparing pH data from HI experiments in piglets with model-simulated pH.

## Experimental Methods and Protocol

All experiments were done under UK Home Office Guidelines (Animals [Scientific Procedures] Act, 1986) and were approved by the Institute of Neurology, University College London. In this study, 1-day-old piglets were ventilated and anaesthetised. Inflatable occluders were surgically placed around the carotid arteries, and arterial partial pressures of oxygen and carbon dioxide, blood glucose and heart rate were maintained at a normal level. Baseline MRS and NIRS were first acquired before transient HI was induced for ~1 h, by inflating the occluders and reducing fractional inspired oxygen (FiO_2_) to 12 % (normal value 21 %). The occluders were released 10–20 min after β-NTP (a correlate of ATP) had reduced by ~70 %, and FiO_2_ was subsequently increased to normalise blood saturation. ^31^P-MRS and broadband NIRS were acquired every 1 min during the baseline period, during HI and for a further ~2 h to monitor recovery from HI [5]. This study is ongoing. As of August 2012, data were obtained from 22 piglets.

NIRS measures changes in the concentrations of oxy- and deoxy-haemoglobin in blood (HbO_2_, HHb). Variations in cerebral blood volume are marked by changes in total (oxy- and deoxy-) haemoglobin concentration. We use MRS (either proton (^1^H) or phosphorus (^31^P)) to observe variations in by-products of cellular metabolism such as inorganic phosphate (Pi), phosphocreatine (PCr), adenosine triphosphate (ATP) and lactate (a marker of anaerobic metabolism). More specifically, we employed ^31^P-MRS to estimate intracellular pH via the chemical shifts of Pi, phosphoethanolamine (PEt) and ATP (pH_Pi_, pH_PEt_ and pH_ATP_ respectively). We have used the following titration curves for pH_Pi_ and pH_PEt_ [5]:

44.1 $${\rm{pH}}_{\rm{pi}}=6.77+{\mathrm{log}}_{10}\left(\frac{{\delta
}_{{\rm{p}}_{\rm{1}}}-3.29}{5.68-{\delta
}_{{\rm{p}}_{\rm{1}}}}\right),\rm{}{\rm{pH}}_{\rm{PEt}}=5.625+{\mathrm{log}}_{10}\left(\frac{{\delta
}_{\rm{PEt}}-3.190}{6.946-{\delta }_{\rm{PEt}}}\right)$$

where δ_Pi_ is the chemical shift difference between PCr resonance and an amplitude weighted mean of the Pi resonances [5]. PEt has been observed to be a major component of the phosphomonoester (PME) peak, and hence, δ_PEt_ is calculated as the chemical shift in PME relative to PCr [5]. Consequently, the mean of pH_Pi_ and pH_PEt_ was adopted as the overall pH_Pi − PEt_ measurement. We used the MAGPAC programme (magnesium and pH from ATP calculation [5]) to calculate pH_ATP_ from the chemical shifts of α-NTP, β-NTP and γ-NTP. In addition, we continuously record systemic variables such as arterial blood pressure (P_a_), arterial oxygen saturation (SaO_2_), breathing rate and heart rate.

## Model

We have developed a mathematical model of blood flow and metabolism, placing emphasis on the physiology of the brain. It consists of a set of algebraic relations and differential equations, describing cerebral blood flow and oxygenation and oxygen and energy metabolism on a cellular level. This system of equations incorporates ~100 parameters and ~25 variables. P_a_, SaO_2_ and arterial carbon dioxide (P_a_CO_2_) are, where available, used as inputs. The model is then able to simulate changes in NIRS-measured HbO_2_ and HHb and MRS-measured Pi, PCr and ATP. It also models changes in the cerebral metabolic rate of oxygen consumption (CMRO_2_) and lactate. Blood flow is modelled as three compartments – arteries and arterioles, capillaries and veins – with varying conductances and radii. We recently added an extra compartment to represent the supply of blood into the arteries [4]. By varying the radius of this new compartment, we can simulate the carotid artery occlusion that results in ischemia. In order to simulate pH in our model, we altered eight reactions as detailed in Table 44.1, to represent the main dynamics of H^+^ions. Changes are shown in bold. Mitochondrial and cytoplasmic protons are modelled separately as H_m_ and H_cyt_. Similarly, we also differentiated between cytoplasmic and mitochondrial NAD and NADH concentrations. p1, p2 and p3 represent the number of protons pumped in each reaction, a3r the concentration of reduced cytochrome a3 and Cu_A,o_ the concentration of oxidised cytochrome-c-oxidase. Cr represents creatine, gluc glucose, Lac lactate, and Py Pyruvate.

**Table 44.1:** Mitochondrial and cytoplasmic reactions modified to simulate intracellular pH

Mitochondria	Cytoplasm
(i) Oxidative phosphorylation $$ \begin{array}{l}2C{u}_{{A}^{\prime }o}+\left(p1+\frac{5}{3}\right){H}_{m}\\ \to 2NAD+\left(p1+4\right){H}_{cyt}\end{array}$$ ${P}_{2}{H}_{m}\to 4C{u}_{{A}^{\prime }o}+4a3r+{p}_{2}{H}_{cyt}$ ${o}_{2}+4C{u}_{{A}^{\prime }o}+{p}_{2}{H}_{m}\to$	(iv) Glycolysis $$ \begin{array}{l}2ADP+2{P}_{i}+gluc+2NA{D}_{cyt}\\ \to 2ATP+2Py+4{H}_{cyt}\end{array}$$
(ii) Tricarboxylic acid cycle $py+5NAD+{H}_{cyt}\to 4{H}_{m}$	(v) PCr to ATP conversion: $PCr+ADP+{H}_{cyt}\to ATP+Cr$
(iii) Protons reenter mitochondria (via leak and complex V): ${H}_{cyt}\to {H}_{m}$	(vi) Pyruvate to lactate conversion $py+{H}_{cyt}\to Lac+NA{D}_{cyt}$

For simplicity, we have kept the total concentrations of cytochrome and NAD at either oxidised or reduced state constant. Therefore, only one oxidisation state is included in the model equations above. We have not modelled the conversion of flavin adenine dinucleotide (FAD) in the TCA cycle, which utilises mitochondrial protons. To compensate, we incorporated 5/3 H_m_ to the left hand side of the oxidative phosphorylation equation (Table 44.1 (i)). On the right hand side of the equation, an additional four protons are pumped into the cytoplasm by complex II. The rate of reaction for glycolysis (Table 44.1 (iv)) was also altered to account for the new reactants. Similar to the mitochondrial proton buffer in the previous model [2], we have added a simple proton buffer in the cytoplasm. Furthermore, we introduced the malate-aspartate shuttle, which transports electrons produced by glycolysis in the cytoplasm across the NADH-impermeable mitochondrial membrane to be used in oxidative phosphorylation. During this enzyme-driven process, NADH in the cytoplasm is oxidised to NAD, while NAD in the mitochondrial matrix is reduced to NADH. We simplified this system and modelled it as a mass action reaction (44.2). The rates for the forward and backward reactions are k_MAshut and k_nMAshut, respectively (Eqs. 44.4 and 44.3). Table 44.2 lists the new parameters that have been added to the model, as necessitated by the changes above.

**Table 44.2:** New parameters and their values

Parameter	Description	Value	Source
Km_glucNN	Km for NAD in the caricature of glycolysis	1.0	[3]
Keq_MAshut	Equilibrium constant for the malate-aspartate shuttle	10.0	[3]
NADcytn	Normal concentration of NAD in the cytoplasm	359	[6]
NADHcytn	Normal concentration of NADH in the cytoplasm	50	[6]

44.2 $$ {H}_{cyt}+NAD\to NA{D}_{cyt}+{H}_{m}$$

44.3 $$ {k}_{-}nMAshut=\frac{{k}_{-}MAshutNADH}{Ke{q}_{-}MAshutNAD{H}_{cyt}}$$

44.4 $$
{k}_{-}MAshut=\frac{\frac{2}{3}CMR{O}_{2r}NAD{H}_{cyt}}{NAD{H}_{cytr}NA{D}_{r}{H}_{cyt}-Ke{q}_{-}MAshu{t}^{-2}\text{}}NA{D}_{cyt}NAD{H}_{r}{H}_{r}$$

## Results

**Fig. 44.1:**
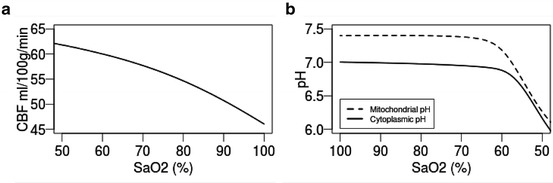
Steady-state model simulations of (**a**) cerebral blood flow (*CBF*) and (**b**) pH against arterial oxygen saturation (SaO_2_)

**Fig. 44.2:**
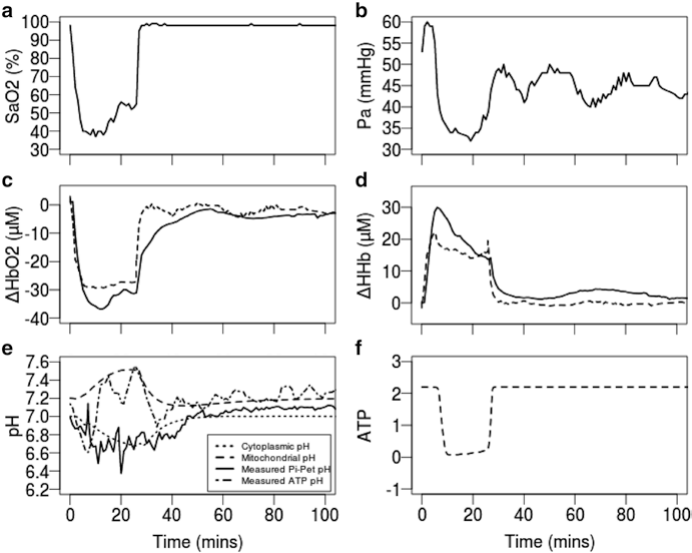
Measured arterial oxygen saturation (SaO_2_, **a**) and blood pressure (P_a_, **b**) used as inputs to the model; NIRS and MRS measurements (*solid line*) from one piglet (LWP180) compared with modelled results (*dashed* and *dotted lines*) (**c–e**); simulated ATP (**f**). pH_ATP_ in (**e**) calculated from MRS measurements of α-NTP, β-NTP and γ-NTP

The steady-state output of the model for cerebral blood flow (CBF) and cytoplasmic and mitochondrial pH with increasing SaO_2_ are illustrated in Fig. 44.1. Normal average brain pH is ~7 [1]. P_a_ and SaO_2_ data from the piglet experiments were used as inputs into the model. PaCO_2_ was not recorded in this instance; however, as the piglets were ventilated with controlled CO_2_ concentrations, we have assumed PaCO_2_ remains constant at 40 mmHg. Due to space constraints, we present in Fig. 44.2 results from only one piglet (LWP180). We used the Morris method to determine the most influential parameters and the SciPy Powell method to detect the optimum values of these parameters to achieve a good fit [7]. Consequently, we increased the values of three parameters in our model; the normal total haemoglobin concentration (Xtot_n) was increased from 5.40 to 6.298 mM, the concentration of cytochrome c oxidase (CCO) in tissue (cytox_tot_tis) from 0.0022 to 0.004257 and the normal oxidised fraction of Cu_A_ (a_frac_n) from 0.67 to 0.75. Other piglets show similar results.

## Discussion

The BrainPiglet model has been extended to simulate the biochemistry affecting intracellular brain pH and shown to successfully predict ^31^P-MRS pH measurements in addition to other metabolic changes. The steady-state simulations (Fig. 44.1) may be used to validate the behaviour of the model. In Fig. 44.1a, the model replicates a common relationship between CBF and SaO_2_, similar to results published earlier [2]. The drop in pH_Pi − PEt_ seen in Fig. 44.1b is indicative of acidosis brought about by a deprivation of oxygen. We have shown successful simulations of metabolic and pH changes in the neonatal brain of one piglet during HI (Fig. 44.2). For a good fit of modelled to measured HbO_2_ and HHb three parameters were optimised – the total concentration of haemoglobin in the blood, CCO and the fraction of blood flowing through the carotid arteries were slightly increased. This implies there is a higher concentration of oxygen supplied to the cell than previously modelled. Such biological parameters may also vary from one individual to another and so can be altered to suit each individual patient. We must note that the comparison of measured and simulated pH is not as straightforward. Although we specifically model intracellular cytoplasmic and mitochondrial pH, ^31^P-MRS provides an average estimate of brain pH in a select area comprised of blood, tissue and various cells. It is not yet possible to clearly distinguish between the different components with this technique of measurement. It may have been anticipated that the modelled cytoplasmic pH fits well with the measured pH_Pi − PEt_ data, as Pi is said to concentrate in the cytoplasm. We also observe in our pH_ATP_ measurement, albeit noisy, an alkaline rise which is concurrent with the modelled mitochondrial pH during HI. These changes occur in tandem with a drop in proton motive force across the mitochondrial membrane – less cytoplasmic protons flow back into the mitochondria, rendering the cytoplasm more acidic.

There are a number of limitations to our model. The oxygen-haemoglobin dissociation curve determines the binding affinity of haemoglobin to oxygen, acting as a biological buffer. However, this rate varies with changes in blood pH; we hope to model this concept in future. In addition, there is possibly a greater variation in pH throughout the brain than that observed in the measurements.

We have effectively modified our model of neonatal brain metabolism and circulation to simulate brain pH. By investigating further, we hope to gain a better understanding of physiological processes during oxygen deprivation. In due course, we aim to adapt the model to the human neonatal brain.
